# Postoperative regular use of a self-rehabilitation mobile application for more than two weeks reduces extension deficit and cyclop syndrome after anterior cruciate ligament reconstruction

**DOI:** 10.1186/s40634-023-00578-z

**Published:** 2023-02-09

**Authors:** Constant Foissey, Hichem Abid, Benjamin Freychet, Bertrand Sonnery-Cottet, Mathieu Thaunat, Jean-Marie Fayard

**Affiliations:** 1 Hôpital Privé Jean Mermoz, Ramsay-Générale de Santé, Lyon, France; 2 Centre Orthopédique Santy, 24 Avenue Paul Santy, 69008 Lyon, France

**Keywords:** Software application, Rehabilitation, Anterior laxity, Fixed flexion deformity, Cyclops syndrome

## Abstract

**Purpose:**

To investigate the minimum use that correlates with the best outcomes in term of complications associated with self-directed rehabilitation mobile application and to explore the user profile and usage habits.

**Methods:**

This was a single-center retrospective study of 356 patients who underwent ACL reconstruction surgery between November 2019 and August 2020. Complications were defined as the presence of an extension deficit ≥ 5° after 6 weeks and/or the presence of cyclops syndrome. The demographics, sports competition level and number of connections were collected by the application.

**Results:**

The complication rate was reduced 4.2-fold with at least 2 weeks of use (2.4% (3/123) (with 0.8% (1/123) of cyclops syndrome) versus 10.8% (23/212) (with 3.3% (7/212) cyclops syndrome), *p* = .04). The mean duration of use was 20 ± 23 days with a frequency of 2.1 ± 2.3 connections per day. The usage rate was 50% in week 1, 35% in week 2, and 24% in week 3. There was one peak in the abandon rate during the first few days of use and a second peak at Day 10 when physiotherapy sessions started. There were two dips in the abandon rate associated with the follow-up visits at Days 21 and 45. Greater use was found in older patients (*p* = .0001) and female patients (*p* = .04).

**Conclusions:**

When using the application for a minimum of 2 weeks, the risk of complications was reduced 4.2-fold. The typical users of a self-directed rehabilitation application after ACL surgery in this study were women and patients over 30 years of age.

**Level of evidence:**

IV, retrospective.

## Background

Orthopedic surgery made the leap into the digital world in the early 2000s. This was felt in the operating room suite with robotics-assisted surgery [1, 2], in the sharing of information, particularly through web sites and social media [3], and increasingly, in the direct contact between patients and their healthcare providers through mobile applications [4]. In the realm of anterior cruciate ligament (ACL) surgery, these specifically help with remote follow-up [5], return to sport [6] and self-directed rehabilitation [7, 8]. Adherence with these programs, their effectiveness, the user profile and their usage habits have not been investigated fully. This information would help us maximize these tools, improve the context and look for ways to improve patient adherence.

Rehabilitation after ACL reconstruction is a key point in the success of this operation [9]. One of the complications of a poorly conducted rehabilitation is to have a persistent extension deficit which itself is at risk of leading to a cyclops syndrome [10] that can reach up to 10.9% of the patients in some series [11]. Among the most recommended re-education program, self-directed rehabilitation plays an essential role [12–15]. This kind of re-education is a favorite topic for mobile applications [7, 16, 17]. In theory, they help to reduce the risk of the patient doing the rehabilitation exercises incorrectly and help to improve the adherence rate to the protocol. A recent study showed the benefits of such an application in the early postoperative course after ACL reconstruction surgery [8]. The aim of this study was to investigate the minimum use that correlates with the best outcomes in terms of complications (persistent extension deficit, cyclops syndrome) and to explore the user profile and usage habits associated with a self-rehabilitation mobile application.

## Methods

A retrospective analysis of data collected prospectively at a single healthcare facility was carried out. All patients who were operated by one of four experienced knee surgeons (JMF, MT, BSC, BF) for ACL reconstruction, had downloaded the *Doct’up®* application between November 2019 and August 2020 and had more than 12 months of follow-up were included. Excluded were patients who had undergone an osteotomy or posterior cruciate ligament, medial/lateral collateral ligament reconstruction. Patients who had undergone meniscectomy, meniscal repair or anterolateral reconstruction (ALR) were not excluded.

### Operative technique

All patients had to fully recover their flexion and extension mobility before the operation. All surgeons used the same technique, in accordance with previously published technique [18–20]. All graft were made with gracilis and semi-tendinosis tendon. The indications for ALR surgery were based on published indications [21]: young age (< 20 years old), participation in pivoting sports or a high-demand athlete, evidence of a high-grade pivot shift on examination, evidence of a lateral femoral notch sign on preoperative imaging, a Segond fracture, chronic (> 12 months) ACL injury.

### Standard rehabilitation

Immediately after surgery, patients were instructed to move their knee between 0–90° for the next 3 weeks. Weightbearing with two crutches was allowed without the use of a splint. The physiotherapy sessions started 10 days after surgery and followed a protocol developed by the surgery team. This protocol is divided in different phases that gradually bring new main objectives: (1) minimize arthrogenic muscle inhibition, re-establish quadriceps control, regain full active extension, (2) maintain full extension, restore full flexion, normalize gait, (3) careful strengthening, (4) strengthening and proprioception, (5) early return to sports, (6) unrestricted return to sports. However, physical therapists were allowed to select exercises and adapt the protocol as needed for a specific patient or provide their own home-based rehabilitation program. The French healthcare system pays for 40 rehabilitation sessions, 30 min in length, following ACL reconstruction.

### Mobile application (Fig. 1)

Patients were introduced to the *DoctUp®* application (Healing SAS, Chassieu, France) during the preoperative visit and told that they could use it in addition to the physiotherapy sessions. This application was created by two surgeons (JMF, MT) and provides a progression of rehabilitation exercises to be used at home between postoperative days 1 and 90. Each exercise is accompanied by a short video providing instructions, describing the goal and potential errors. It is available for free on the Google Play Store and the Apple App Store. To be considered a user, the patient must have used the application at least once every 2 days during its period of use; as a use higher than this treshold would correspond to the minimum frequency recommended in the unit of kinesitherapy sessions (at least one every three days).

### Data collection

When they first logged into the application, patients completed a short questionnaire that captured their demographics (age, sex, height, weight), their sports competition level (beginner, intermediate, advanced) and their surgery date. The application console provides information about the date of each connection to the application by every patient, along with the exercises done. This allowed us to evaluate a patient's usage pattern. All patients were aware of the use of their data and gave their consent.

Clinical follow-up visits with the medical team occurred on postoperative day 21, day 45, month 3, month 6 and at the end of 1 year. At each visit, the range of motion (ROM) was measured, and any complications documented. Significant knee extension deficit was defined as extension deficit ≥ 5° measured with a long arm goniometer [22]. Complications were defined as the presence of an extension deficit ≥ 5° after 6 weeks and/or the presence of cyclops syndrome within the follow-up period. An MRI was performed to every patient with a persistent knee extension deficit after 3 months. The authors focused on the presence of an extension deficit at 6 weeks, as this is most predictive of the development of cyclops syndrome [10]. Cyclop syndrome was defined as a persistant loss of full knee extension due to the development of a fibrous nodule at the base of the ACL confirmed on MRI [11].

### Statistical analysis

Quantitative variables were expressed as their mean ± SD [minimum; maximum]. Qualitative variables were expressed as percentages. The user profile was investigated using an analysis of covariance to analyze the last day of use as a function of sex, age (< vs ≥ 30 years-old) and sports competition level. Factors contributing to complications (i.e., cyclops syndrome and extension deficit at 6 weeks) were analyzed using a binomial logistic regression with the variables age, sex, competition level, and application usage time (< 7 days, 7–13 days, 14–20 days, ≥ 21 days). The significance threshold was set at p ≤ 0.05. All these analyses were done with XLSTAT® software (Addinsoft (2021), Paris, France).

## Results

Three hundred fifty-six patients met the inclusion criteria; 21 were lost to follow-up and excluded from the analysis. The mean follow-up was 17 ± 3 [1, 3, 5, 11, 13, 14, 17, 22–26] months. The demographic data are summarized in Table 1.

**Table 1:** Patient demographics and usage statistics

	Population (*n* = 335)
Number of connections	59 ± 12 [0–1289]
Last connection (days postoperative)	20 ± 23 [0–90]
Frequency of use (connections/day):	
Total	2.1 ± 2.3 [0–14]
D0–D10	2.7 ± 2.1 [0–31]
D11–D21	3.5 ± 3.7 [0–23]
D22–D45	3.0 ± 3.3 [0–15]
> D45	2.0 ± 1.4 [0–8]

*D* Days

**Fig. 1:**
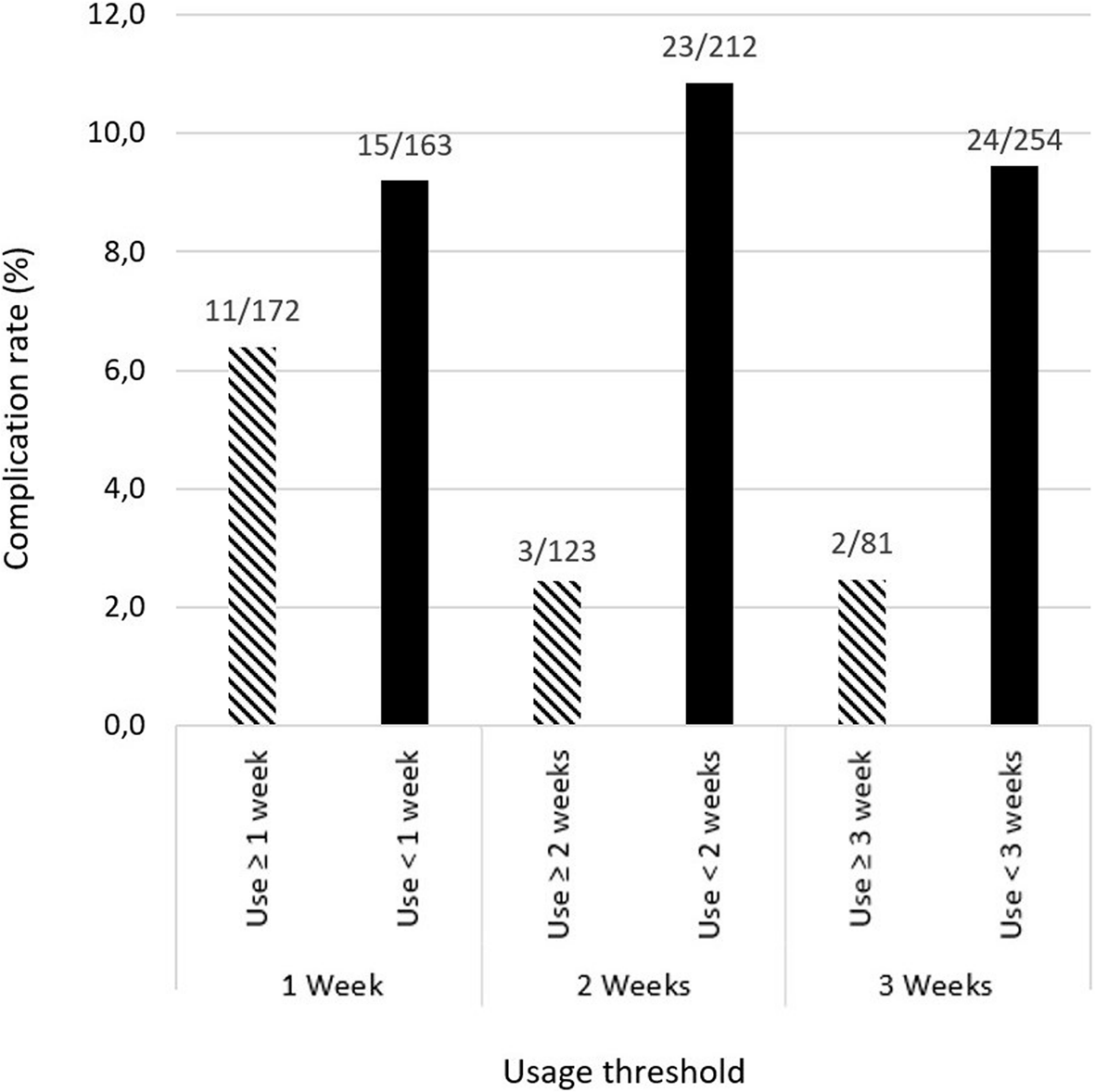
Complication rate as a function of the preset usage threshold

Overall, 22 patients (6.6%) had an extension deficit at 6 weeks. At the final assessment, there were eight instance of cyclops syndrome (2.4%) and among these patients, 4 (50%) had an extension deficit at 6 weeks. The multivariate analysis found that the usage rate was a protective factor against complications (OR = 0.24 for more than 2 weeks of use; OR = 0.25 for more than 3 weeks of use, *p* = 0.04) (Table 2). This benefit was obvious with as little as 2 weeks of use, with the complication rate dropping to 2.4% (3/123) in the app user group (0.8% (1/123) cyclops syndrome) versus 10.8% (23/212) in the non-user group (3.3% (7/212) cyclops syndrome) (*p* = 0.005) (Fig. 1).

**Table 2:** Multivariate analysis of complications

	No complications (*n* = 309)	Complications (*n* = 26) (Cyclops lesion/extension deficit at 6 weeks)	OR	*P* value
Age (years)	31 ± 11 [11–67]	30 ± 8 [15–46]	0.86	.92
Sex (female)	137 (44%)	11 (42%)	1	.92
Competition level				.94
- Beginner	34 (11%)	3 (12%)		
- Intermediate	152 (49%)	13 (50%)	1.1	
- Advanced	124 (40%)	10 (38%)	0.95	
Usage				**.04**
< 7 days	148 (48%)	15 (58%)		
7–13 days	41 (13%)	8 (31%)	1.9	
14–20 days	41 (13%)	1 (4%)	0.24	
≥ 21 days	79 (25%)	2 (7%)	0.25

**Fig. 2:**
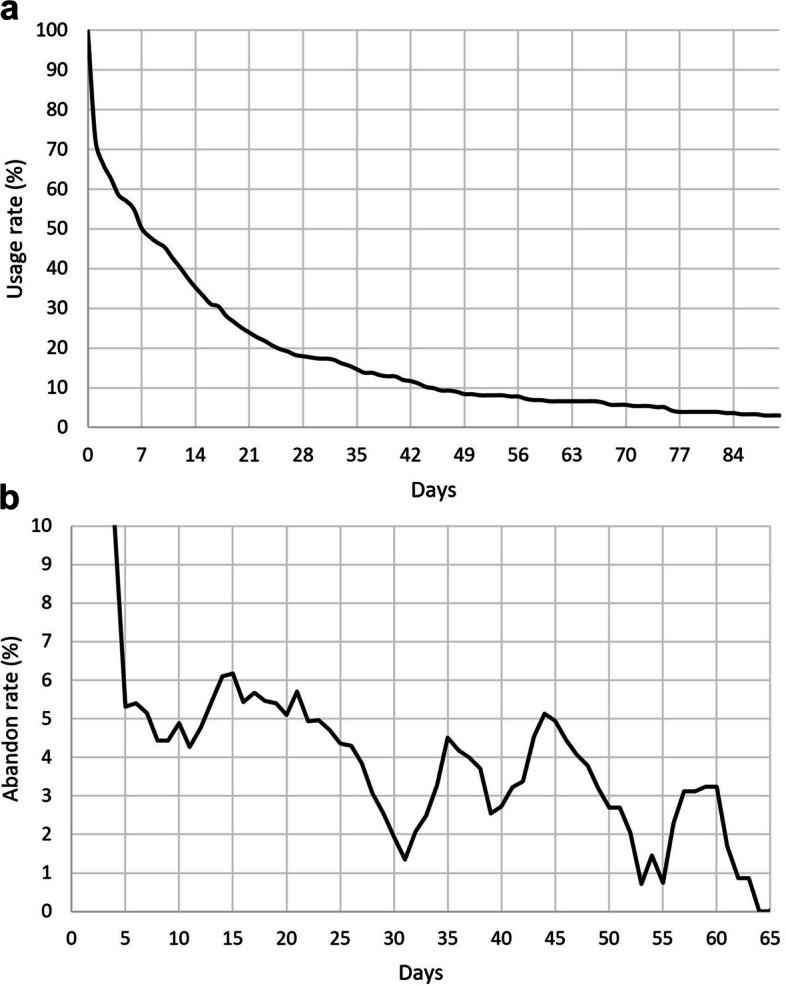
Usage rate (2**a**) and abandon rate (5 day moving average) (2**b**) of the application during the postoperative course

On average, patients used the app up to the 20^th^ day ± 23 with a frequency of 2.1 ± 2.3 connections per day; this frequency of use was consistent over time (Table 3). The usage rate was 50% in week 1, 35% in week 2, and 24% in week 3 (Fig. 2a). The abandon rate peaked at Day 10 when physiotherapy sessions started. There were two dips in the abandon rate associated with the follow-up visits at Days 21 and 45 (Fig. 2b).

**Table 3:** User profile

	Population (*n* = 335)
Age (years)	30 ± 11 [11–67]
Sex (female)	148
Competition level	
- Beginner	37 (11%)
- Intermediate	165 (49%)
- Advanced	134 (40%)
BMI (kg/m^2^)	24 ± 23 [17–44]

The covariance analysis found that the usage rate increased with age (17 ± 20 days of use in those under 30 years versus 24 ± 25 in those over 30 years, *p* = 0.0001) and was higher in women than men (23 ± 19 days of use versus 18 ± 15, *p* = 0.04). The competition level was not significantly related to the usage pattern (*p* = 0.17).

## Discussion

The most important finding of this study is that when using regularly a self-directed rehabilitation application postoperatively for a minimum of 2 weeks, the risk of complications was reduced 4.2-fold; 35% of the patients met this threshold. The typical users of this application after ACL surgery in this study were women and patients over 30 years of age.

The early extension deficit of the knee is a mechanism against which it is important to fight, not only because it is an important risk factor of cyclop syndrome [10], but also because it causes an increase of the loads at the lumbar level [24], a poorly tolerated gait disturbance, an increase of the loads with regard to the patella responsible for knee pain and being able to give a premature cartilage degeneration of the medial compartment [26, 27]. The 4.2-fold reduction in complications after a minimum of 2 weeks of use is a strong argument in favor in increasing adherence. These results reinforce those of a recent study showing less pain and better quadriceps activation during walking at the 3-week follow-up in patients who used an application like this one [8]. To the authors’ knowledge, this was the only study to investigate a mobile application for rehabilitation after ACL reconstruction. The effectiveness against extension deficit and cyclops lesions can be explained by the emphasis placed on waking up the quadriceps and re-establishing the gait pattern in the early stages of the application’s use, with exercise based on combatting the muscle inhibition that can contribute to arthritis [25, 28]. In a recent meta-analysis, the rate of cyclops syndrome was found to range from 1.9% to 10.9% [11]. While not directly comparable, in this study, were found that patients who used the application for at least 2 weeks had a relatively low rate of cyclops syndrome (0.8%), whereas patients who used the application for less than 2 weeks were in the typical range (3.3%).

The adherence rate to a standard self-directed rehabilitation program has not been reported in the literature; while the reported usage rate here appears to be low, there is no comparators. This is despite the fact that this sector is undergoing rapid growth; in 2015, there were already 165,000 apps in the healthcare domain [4]. In 2019, 76 of them were focused solely on sports medicine [23]. This detailed analysis of usage patterns will help us identify ways to improve the adherence. The highest abandon rate was during the first week, with half the patients downloading the app but not using it. Better patient education preoperatively and immediate postoperatively should help to reduce this initial loss of users. The second peak in the abandon rate was around day 10, which corresponds to the start of physiotherapy sessions in the protocol. Consequently, a better communicate with physical therapists is needed about the benefits of this application in combination with in-person sessions. Two dips in the abandon rate following the 21-day and 45-day postoperative follow-up visits were also seen, evidence that the patients are open to the encouragement provided by their surgeon or sports medicine physician. Two ways to ensure patient adherence can be set up: first, more regular notifications in the app when the usage rate starts to drop; second, creation of a telemonitoring platform that will allow healthcare providers (surgeons, physicians, physical therapists) to follow their patient’s involvement. Lastly, the analysis of the typical user profile suggests that therapeutic patient education must be focused on males under 30 years of age.

This study has several limitations. First, data such as education level and occupation that would have helped better understand the user profile were not available. Since this was not a randomized study, there may have been a selection bias for patients using the application; the high users may have been highly motivated patients who would have done well even without the app. Lastly, while a detailed analysis of usage statistics was available, it is impossible to account for patients who did the exercises described in the app without regularly logging on.

## Conclusion

When using the self-directed rehabilitation application after ACL surgery for a minimum of 2 weeks, the risk of complications was reduced 4.2-fold; 35% of the patients met this threshold. The typical users of the application were women and patients over 30 years of age. A detailed analysis of the user profile will help us target at-risk patients and convince them to be more active participants in their care.

## Data Availability

Not Applicable.
